# A loss-of-function mutation p.T52S in *RIPPLY3* is a potential predisposing genetic risk factor for Chinese Han conotruncal heart defect patients without the 22q11.2 deletion/duplication

**DOI:** 10.1186/s12967-018-1633-1

**Published:** 2018-09-21

**Authors:** Nanchao Hong, Erge Zhang, Qingjie Wang, Xiaoqing Zhang, Fen Li, Qihua Fu, Rang Xu, Yu Yu, Sun Chen, Yuejuan Xu, Kun Sun

**Affiliations:** 1 Department of Pediatric Cardiology, Xinhua Hospital, Affiliated to Shanghai Jiao Tong University School of Medicine, Room 505, Scientific Building, Shanghai, 200092 China; 2 Scientific Research Center, Xinhua Hospital, Affiliated to Shanghai Jiao Tong University School of Medicine, Shanghai, 200092 China; 3 Department of Pediatric Cardiology, Shanghai Children’s Medical Center, Affiliated to Shanghai Jiao Tong University School of Medicine, Shanghai, 200127 China; 4 Medical Laboratory, Shanghai Children’s Medical Center, Affiliated to Shanghai Jiao Tong University School of Medicine, Shanghai, 200127 China

**Keywords:** Conotruncal heart defect, RIPPLY3, Target sequencing, TBX1

## Abstract

**Background:**

Conotruncal heart defect (CTD) is a complex congenital heart disease with a complex and poorly understood etiology. The transcriptional corepressor RIPPLY3 plays a pivotal role in heart development as a negative regulator of the key cardiac transcription factor TBX1. A previous study showed that *RIPPLY3* contribute to cardiac outflow tract development in mice, however, the relationship between *RIPPLY3* and human cardiac malformation has not been reported.

**Methods:**

615 unrelated CTD Chinese Han patients were enrolled, we excluded the 22q11.2 deletion/duplication using a modified multiplex ligation-dependent probe amplification method—CNVplex^®^, and investigated the variants of *RIPPLY3* in 577 patients without the 22q11.2 deletion/duplication by target sequencing. Functional assays were performed to testify the potential pathogenicity of nonsynonymous variants found in these CTD patients.

**Results:**

Four rare heterozygous nonsynonymous variants (p.P30L, p.T52S, p.D113N and p.V179D) were identified in four CTD patients, the variant NM_018962.2:c.155C>G (p.T52S) is referred as rs745539198, and the variant NM_018962.2:c.337G>A (p.D113N) is referred as rs747419773. However, variants p.P30L and p.V179D were not found in multiple online human gene variation databases. Western blot analysis and immunofluorescence showed that there were no significant difference between wild type *RIPPLY3* and these four variants. Luciferase assays revealed that the p.T52S variant altered the inhibition of TBX1 transcriptional activity in vitro, and co-immunoprecipitation assays showed that the p.T52S variant reduced the physical interaction of RIPPLY3 with TBX1. In addition to the results from pathogenicity prediction tools and evolutionary protein conservation, the p.T52S variant was thought to be a potentially deleterious variant.

**Conclusion:**

Our results provide evidence that deleterious variants in *RIPPLY3* are potential molecular mechanisms involved in the pathogenesis of human CTD.

**Electronic supplementary material:**

The online version of this article (10.1186/s12967-018-1633-1) contains supplementary material, which is available to authorized users.

## Background

Congenital heart disease (CHD), including heterogeneous anatomy with distinct phenotypic subtypes, is the most common form of birth defect in humans, and it affects approximately 1% of all live births [1, 2]. Among these, conotruncal heart defect (CTD) is a severe malformation. It is characterized by a disordered orchestration of the ventricles, the aorta and the pulmonary artery, and consists of the tetralogy of Fallot (TOF), transposition of the great arteries (TGA), double outlet of right ventricle (DORV), persistent truncus arteriosus (PTA), pulmonary atresia with ventricular septal defect (PA/VSD), and interrupted aortic arch (IAA). CTD is estimated to occur in approximately 1 out of 1000 live births [3]. It is the most common cyanosis CHD, and it usually requires surgical treatment. Patients born with CTD often need lifelong specialized cardiac care.

Although our understanding of the molecular pathways underlying cardiac development has grown remarkably in the past few years, most genetic factors that predispose patients to CTD remain unidentified [4]. Most of the known causes of CTD are sporadic genetic changes such as nucleotide variants or focal copy-number DNA variations [4]. 22q11.2 deletion syndrome is the most common microdeletion syndrome, affecting nearly 1 in 2000–4000 live births [5]. It has been reported to be associated with various types of congenital heart defects, especially CTD [6]. *TBX1* (MIM 602054), a member of the phylogenetically conserved T-box gene family of DNA-binding transcription factors, was mapped to the critical 1.5 Mb deletion region of 22q11.2 [4]. *TBX1* variants were reported to be responsible for the cardiac phenotype of 22q11.2 deletion syndrome, and it plays a vital role in the development of the cardiac outflow tract [7–11]. However, copy-number or single-nucleotide variants of *TBX1* account for only approximately 7% of CTD patients in China [11, 12].

RIPPLY proteins (including RIPPLY1/2/3) have been shown to modulate the transcriptional properties of T-box proteins, which play a pivotal role in embryonic development [13–16]. They interact with the transcriptional corepressor Groucho/TLE and the T-box proteins through two distinct amino acid sequences: the WRPW motif, which is a highly conserved four-amino-acid stretch in the N-terminal half, and the Ripply homology (RH) domain, which is a conserved ~ 50-amino-acid stretch that interacts with the T-domain [15, 16]. In mouse embryo, *RIPPLY1* mRNA was first detected at E8.5, and was continuously expressed until E12.5. During this period, *RIPPLY1* was mainly expressed in the anterior presomitic mesoderm (PSM) [17]. Similarly, *RIPPLY2* expression was localized to the PSM in wild-type mouse embryos from E9.0 to E11.5, when somites were being formed [18]. Moreover, *RIPPLY2*-null mice had a partial pedicle of neural arches and separated lamina, *RIPPLY1/2* double-null mice showed a complete loss of pedicle and fusion of the lamina in the vertebrae [17]. In ripply1-deficient zebrafish embryos, somite boundaries do not form, and the characteristic gene expression in the presomitic mesoderm (PSM) is not properly terminated [19]. These results revealed that *RIPPLY1* and *RIPPLY2* play important roles somite segmentation during development [17–20].

*RIPPLY3*, also known as Down syndrome critical region 6 (*DSCR6*; MIM 609892), plays a critical role in the development of cardiac outflow tract. Previous studies suggested that *Ripply3* knockout appeared to lead to the abnormal development of the cardiac outflow tract, including hypotrophy of the aorta and incomplete formation of the ventricular septum in mice [15]. Further evidence showed that RIPPLY3 can repress the intrinsic transcriptional property of TBX1 by recruiting the Groucho/TLE co-repressor [15]. TBX1 loss- and/or gain-of-function in mice led to malformations of the cardiac outflow tract [21–23], partly resembling the *Ripply3*-deficient embryos. However, the relationship between *RIPPLY3* and human cardiac malformation has not been investigated.

Since copy number or single-nucleotide variants of *TBX1* account for only a small proportion of CTD cases, and its corresponding protein partner *RIPPLY3* is also required for normal development of cardiac outflow tract, we hypothesized that deleterious variants in *RIPPLY3* gene may be potential molecular mechanisms involved in the pathogenesis of human CTD. Therefore, we screened for *RIPPLY3* variants in a CTD cohort and assessed potentially deleterious variants. Here, we report four rare nonsynonymous *RIPPLY3* variants in four CTD patients. We also show that the *RIPPLY3* variant p.T52S confers consistent functional changes. This is the first report of a human CHD phenotype related to genetic variation in *RIPPLY3.*

## Methods

### Gene nomenclature information

The *RIPPLY3* sequence used in this work is accessible as NCBI RefSeq NM_018962.2. Nucleotide numbering was based on the cDNA sequence (#NM_018962.2) with + 1 corresponding to the A of the ATG initiation codon of translation (exon 1) in the reference sequence.

### Ethics statement

All assessments were done with the approval of the Medical Ethics Committee of Xinhua hospital and Shanghai Children’s Medical Center. And all experiments were carried out in accordance with the approved guidelines. Fully written informed consent was obtained from all participants (or their parents if children were too young to consent by themselves).

### Study subjects and DNA isolation

From November 2011 to January 2014, a cohort of 615 unrelated patients of Han ethnicity diagnosed with CTD were recruited from Shanghai Children’s Medical Center affiliated to Shanghai Jiao Tong University School of Medicine, a cardiologist confirmed the CHD diagnosis for all patients by reviewing and evaluating patient history, physical examinations, and medical records (Table 1) [12]. All subjects were unrelated, and the median age at the time of diagnosis was 10 months with a range of 3 days to 18 years. A total of 391 unrelated healthy individuals (median age was 3.5 years), free of CHD, of Han ethnicity were also enrolled as controls. Peripheral blood samples of all cases were obtained, and genomic DNA was extracted using the QIAmp DNA Blood Mini Kit (Qiagen, Hilden, Germany) with standard protocols.

**Table 1:** Cardiac diagnoses for study cohorts

Diagnosis	Number	Percentage
TOF	231	37.6
PA + VSD	135	21.9
DORV	115	18.7
TGA	91	14.8
IAA	11	1.8
TA	20	3.3
PTA	12	1.9
Total	615	100

TOF tetralogy of Fallot, PA + VSD pulmonary atresia with ventricular septal defect; DORV double outlet of right ventricle, TGA transposition of the great arteries, IAA interrupted aortic arch, TA tricuspid atresia, PTA persistent truncus arteriosus

22q11.2 loci in all CTD patients were explored by CNVplex^®^ (a technique for high-throughput detection of sub-chromosomal copynumber aberrations) as described before [12]. Finally, 577 patients without 22q11.2 deletion/duplication were recruited.

### Genetic analysis

For the patients without 22q11.2 deletion/duplication, fragments covering the promoter region, 5′UTR, coding sequence and splicing site of *RIPPLY3* were amplified using the EasyTarget^®^ amplification kit (Genesky Biotechnologies Inc, Shanghai, China) which was developed according to the cycled primer extension and ligation-dependent amplification (CPELA) method. The CPELA was a new method developed by Genesky Biotechnologies for fast and simple enrichment of multiple gene regions for massively parallel sequencing [24].

All candidate variants of *RIPPLY3* were confirmed by Sanger sequencing. The primer pairs used to amplify the coding regions contain candidate variants were designed using Primer Premier 5 in Additional file 1: Table S1. Samples were amplified in a volume of 10 μl containing 1× MyTaq™Mix (Bioline USA Inc.), 200 ng of DNA and 0.4 μM of each primer following the suggested PCR protocol. PCR products were sequencing on an ABI 3730 sequencer (Applied Biosystems, USA). The PCR program was as follows: 98 °C for 1 min; 30 cycles of 98 °C for 20 s, 62 °C for 30 s and 72 °C for 30 s; then 72 °C for 10 min. Amplified products were analyzed on 1% agarose gels stained with GelRed (Biotium, USA). The sequence traces were aligned with the reference sequence for *RIPPLY3* (RefSeq NM_018962.2) using the GenBank BLAST program (http://blast.ncbi.nlm.nih.gov/Blast.cgi).

Furthermore, because mutations in cardiac kernel members, such as *GATA4*, *NKX2*-*5*, and *TBX5*, underlie a range of CHDs [25, 26], and *TBX1* plays a vital role in the development of the cardiac outflow tract [7–11]. To validate the potential function of identified *RIPPLY3* variant, we also screened coding sequences and splicing sites of these known CHD pathogenic genes (*TBX1*, *GATA4*, *NKX2.5* and *TBX5*) in CTD patients and controls using the EasyTarget^®^ amplification kit (Genesky Biotechnologies Inc, Shanghai, China). All candidate variants were also validated by Sanger sequencing. The primer pairs used to amplify the coding regions contain candidate variants were designed using Primer Premier 5 in Additional file 1: Table S2. The verified sequence variants were queried in the SNP database at National Center for Biotechnology Information (NCBI; http://www.ncbi.nlm.nih.gov), 1000 Genomes database (http://www.1000genomes.org), Exome Aggregation Consortium database (ExAC, http://exac.broadinstitute.org) and Ensembl database (http://asia.ensembl.org).

### In silico analysis

To predict the potential pathogenic impact of missense variants, three different pathogenicity prediction tools (PPTs), namely PolyPhen2, SIFT and Mutation Taster were used.

### Alignment of multiple RIPPLY3 protein sequences

Multiple RIPPLY3 protein sequences across various species were aligned using the clustalx 2.1 software.

### Plasmid constructs

*RIPPLY3* expression vector pCMV6-Entry-RIPPLY3 (Myc-DDK-tagged) containing the cDNA of human *RIPPLY3* (RefSeq NM_018962.2) was purchased from Origene (Rockville, MD, USA). The identified variants were introduced into wild type pCMV6-Entry-RIPPLY3 using a QuickChange II Site-Directed Mutagenesis kit (Stratagene, La Jolla, CA, USA). Both wild type and variant inserts were sequenced by Sanger sequencing to confirm the variant sequence and exclude any other sequence variations. *TBX1C* expression vector pCMV6-XL6-TBX1 containing the cDNA of human *TBX1C* (RefSeq NM_080647.1) was purchased from Origene (Rockville, MD, USA). *TBX1C* cDNA was digested and inserting into the plasmid pcDNA3.1(+) at the *Kpn*I and *Xho*I sites. The luciferase reporter *Wnt5a*-luc, was constructed by inserting the conserved T-box binding sites (TBEs) in the 3′-UTR of human *WNT5A*, a target of *TBX1* [27], into the pGL3-promoter plasmid (Promega, USA). An *FGF10* luc reporter was also constructed according to the described by Agarwal et al. [28]. *FGF10* genomic sequence was obtained from the GenBank (http://www.ncbi.nlm.nih.gov). A fragment comprising 4.5 kb upstream of the coding region (− 4.3 kb/+ 176 bp) of *FGF10* was amplified from DNA of a healthy individual and inserted into the *Mlu*I/*Sma*I sites of the pGL3-Basic vector (Promega, Madison, Wisconsin, USA) to generate the *FGF10*-luc reporter construct. The schemes of these two luciferase reporter plasmids were showed in Fig. 3b, c.

### Cell culture and transfection

HEK293T and C2C12 cell lines were purchased from the Type Culture Collection of the Chinese Academy of Sciences (Shanghai, China) and cultured in the DMEM medium (Invitrogen, California, USA) supplemented with 10% foetal calf serum (Invitrogen, California, USA), penicillin (100 units/ml) and streptomycin (100 μg/ml). Cells were incubated at 37 °C with 5% CO_2_. All the transient transfections were performed with Fugene HD transfection reagent (Promega, Madison, Wisconsin, USA) according to the manufacturer’s protocol for adherent cells.

### Dual luciferase reporter assay

HEK293T or C2C12 cells were seeded into 48-well plates 24 h before transfection. Plasmids including the wild-type or variant RIPPLY3 vector, the TBX1 vector, the luciferase reporters and the corresponding renilla luciferase reporter were co-transfected into HEK293T or C2C12 cellsusing Fugene HD following the manufacturer’s protocol. Transfected cells were incubated for 36 h, then washed using DPBS and lysed using 1× passive lysis buffer for 15 min at room temperature (RT) provided by the Dual Luciferase Reporter Assay Kit (Promega). Firefly luciferase and renilla luciferase activities were measured with the Dual-Glo luciferase assay system (Promega) and the Centro XS^3^ LB 960 Microplate Luminometer (Berthold, Bad Wildbad, Germany) according to the manufacturer’s recommended protocol. The transfection efficiency was normalized to paired renilla luciferase activity. The results are the means of three independent experiments, each done in duplicate.

### Immunofluorescence

HEK293T cells were seeded into 12-well plates 24 h before transfection, and cells were transiently transfected with a total 800 ng pCMV6-Entry-RIPPLY3 or variant RIPPLY3 constructs, and pcDNA3.1-TBX1 was also transfected as positive control. Cells were harvested 24 h after transfection, washed three times with PBS, fixed using 4% paraformaldehyde/PBS for 10 min and washed again in PBS. Cells were permeabilized using 0.3% Triton X-100/PBS for 10 min. After washing with PBS, the cells were blocked with 5% BSA/PBS solution for 1 h at RT. Then, the cells were incubated with a 1:100 solution of the rabbit primary anti-TBX1 antibody (ab18530, abcam) or 1:200 solution of the goat primary anti-Myc tag antibody (ab9132, abcam) in 1% BSA/PBS overnight at 4 °C. Next,the cells were washed with PBS and incubated with a 1:500 solution of the secondary anti-Rabbit IgG H&L (Cy3^®^) (ab6939, abcam) or 1:200 solution of the secondary Anti-Goat IgG H&L (Alexa Fluor^®^ 488) preadsorbed (ab150129, abcam) in 1% BSA/PBS for 2 h at RT. After washing with PBS, the cells were incubated with DAPI for 6–8 min at RT, then washed with PBS and mounted on a rectangular slide containing an anti-fading agent. The slides were examined using the Olympus BX43 microscope (Olympus, Shinjuku-ku, Tokyo, Japan).

### Western blot

To confirm whether these variants affect the expression of RIPPLY3 protein, western blot analysis was carried out. HEK293T cells were transfected with a total of 500 ng of pCMV6-Entry-RIPPLY3 or variant RIPPLY3 constructs in 24-well plates. Cells were harvested 40–48 h after transfection and lysed in 90 μl cell lysis buffer. After boiling for 10 min, cell lysates were analyzed by sodium dodecyl sulfatepolyacrylamide gel electrophoresis and electrotransferred to Nitrocellulose Blotting Membrane (GE Healthcare). Then, we block the membranes with 5% skim milk for 2 h at RT, and incubated the membranes with anti-Myc tag antibody (ab9132, abcam) and anti-actin antibody (ab3280, abcam) overnight at 4 °C. Next, the membranes were incubated with horseradish peroxidase-conjugated secondary antibodies (Proteintech, Chicago, IL, USA) for 2 h at RT. The membranes were washed, and detected using enhanced chemiluminescence (ECL) reagents (Millipore, Billerica, MA, USA). Bands were visualized with a chemiluminescence detection system (BioRad, Philadelphia, PA, USA) and analyzed using the Imagelab program (BioRad, Philadelphia, PA, USA).

### Co-immunoprecipitation assay

For co-immunoprecipitation experiment, HEK293T cells were cotransfected with a total 7.2 μg pcDNA3.1-TBX1 and pCMV6-Entry-RIPPLY3 or variant RIPPLY3 constructs. The washed Protein A/G Sepharose (GE Healthcare) was mixed with 6 μg of rabbit anti-TBX1 antibody (ab18530, abcam) and placed on a shaker at 4 °C for 3 h. Then, 1 mg protein from HEK 293T cell lysates were added and left at 4 °C for 8 h or overnight. After washing 4 times with PBS, the pellets were resuspended in 30 μl 1× Protein Loading buffer (Sangon Biotech), boiled for 10 min at 100 °C, and the products were analyzed by sodium dodecyl sulfatepolyacrylamide gel electrophoresis, next, a regular Western blot was run as previously described. The grayscale value of protein bands was calculated using Image J software.

### Statistical analysis

Results are expressed as mean ± standard deviation. The independent-samples t-test was used to determine statistical significance of unpaired samples. The Chi square test was used to determine statistical significance of allele frequency between CTD patients and controls. The two-sided statistical tests were considered significant with a level of *p *< 0.05. All statistical analysis in our study was carried out with SPSS 22.0 (SPSS Inc. Chicago, IL, USA).

## Results

### Four missense variants of *RIPPLY3* were identified in 22q11.2 CNV-negative patients

Four heterozygous missense variants in *RIPPLY3* were identified in 4 unrelated CTD patients out of the 577 CTD patients without 22q11.2 deletion/duplication, all the variants were absent from the 391 controls in our cohort: p.P30L in TOF, p.T52S in TOF, p.D113N in TGA/PA/VSD, and p.V179D in PA/VSD and patent ductus arteriosus (PDA) (Table 2).

**Table 2:** Detailed information of missense variants identified in RIPPLY3

Patient ID	Variants		Status	Diagnosis	SIFT score	PloyPhen V2 Score	Mutation Taster
	NP_061835.1	NM_018962.2					
F029	p.P30L	c.89C>T	Unreported	TOF	0.01	0.35	p
F166	p.T52S	c.155C>G	rs745539198	TOF	0.17	0.79	p
A002	p.D113N	c.337G>A	rs747419773	TGA/VSD/ASD/PS	1.00	0.003	p
PI011	p.V179D	c.536T>A	Unreported	PA/VSD/PDA	0.71	0	p

TOF tetralogy of Fallot, TGA transposition of the great arteries, VSD ventricular septal defect, ASD atrial septal defect, PS pulmonary stenosis, PA/VSD pulmonary atresia with ventricular septal defect, PDA patent ductus arteriosis, p polymorphism

**Fig. 1:**
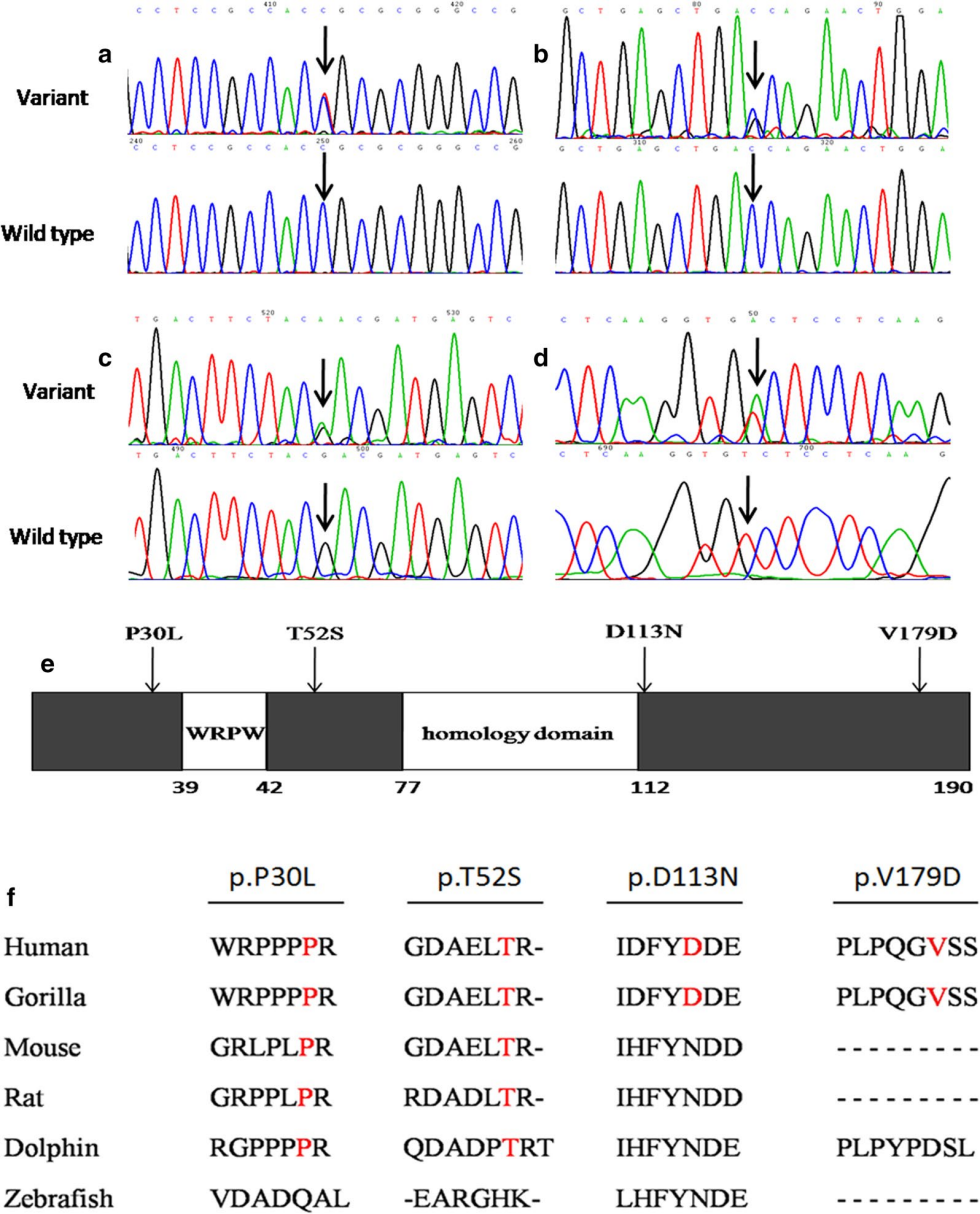
RIPPLY3 variants in CTD patients. **a**–**d** Chromatograms of the RIPPLY3 variants found in CTD patients. The arrow indicates the heterozygous nucleotides of C/T (**a**), C/G (**b**), G/A (**c**) or T/A (**d**) in four CTD patients, or the homozygous nucleotides of C/C (**a**), C/C (**b**), G/G (**c**) or T/T (**d**) in the control individuals (wild-type); **e** structural representations of the variants in RIPPLY3 protein. **f** Alignment of multiple RIPPLY3 protein sequences among species. The altered amino acids of P30 and T52 are shown to be highly conserved evolutionarily across various species

Within the group of 4 missense RIPPLY3 variants, p.T52S (NM_018962.2:c.155C>G, rs745539198) and p.D113N (NM_018962.2:c.337G>A, rs747419773) were found in ExAC database, the allelic frequencies of these two variants were 3.32e−05 and 1.654e−05 respectively, and the p.T52S variant was found only in Asian population. Notably, variants p.P30L and p.V179D were not identified in the NCBI’s SNP database, 1000 Genomes database, ExAC database or Ensembl database. The chromatograms showing the identified heterozygous *RIPPLY3* variants in contrast to corresponding control sequences are shown in Fig. 1a–d, and the positions of these variants in the RIPPLY3 protein are illustrated in Fig. 1e.

As shown in Fig. 1f, a cross-species alignment of multiple RIPPLY3 protein sequences showed that both the p.P30L and p.T52S occur in the highly evolutionarily conserved residues, indicating that the amino acids are functionally important. However, the valine 179 of the protein was just conserved in humans and gorillas, and the aspartate 113 of the protein was not conserved.

Several known CHD pathogenic genes (*TBX1*, *GATA4*, *NKX2.5* and *TBX5*) were also screened in these four patients. There were no nonsynonymous variants identified in *GATA4*, *NKX2.5* and *TBX5*, but two known nonsynonymous single nucleotide polymorphisms (SNPs) were identified in *TBX1C* (Table 3). The nonsynonymous SNP rs72646967 (*TBX1C* NM_080647.1:c.1189A>C) appeared commonly in population (MAF > 0.05), and the allele frequency was similar between CHD patients and controls [29]. The another nonsynonymous SNP rs41298838 (*TBX1C* NM_080647.1:c.928G>A) was previously predicted to be a tolerant polymorphism [30], and was found in both the non-del22q11 CTD patients and healthy controls at similar frequencies [31]. The allele frequency of rs72646967 and rs41298838 was similar between CTD patients and controls in this study (p > 0.05) (Table 3). Therefore, there were no deleterious variants identified in these four CHD pathogenic genes (*TBX1*, *GATA4*, *NKX2.5* and *TBX5*) in patients harboring *RIPPLY3* variants.

**Table 3:** TBX1 variants identified in patients harboring RIPPLY3 variants

Patients ID	RIPPLY3 variants NP_061835.1	TBX1C variants			TBX1C variants allele frequency		
		NM_080647.1	NP_542378.1	SNP	CTD (n = 577)	Control (n = 361)	p value
F029	p.P30L	c.1189A>C	p.N397H	rs72646967	0.521	0.495	0.39
F166	p.T52S	–	–	–	–	–	–
A002	p.D113N	c.1189A>C	p.N397H	rs72646967	0.521	0.495	0.39
PI011	p.V179D	c.928G>A	p.G310C	rs41298838	0.0561	0.0556	0.95

CTD conotruncal heart defect

### The variants do not affect the intracellular localization of RIPPLY3

**Fig. 2:**
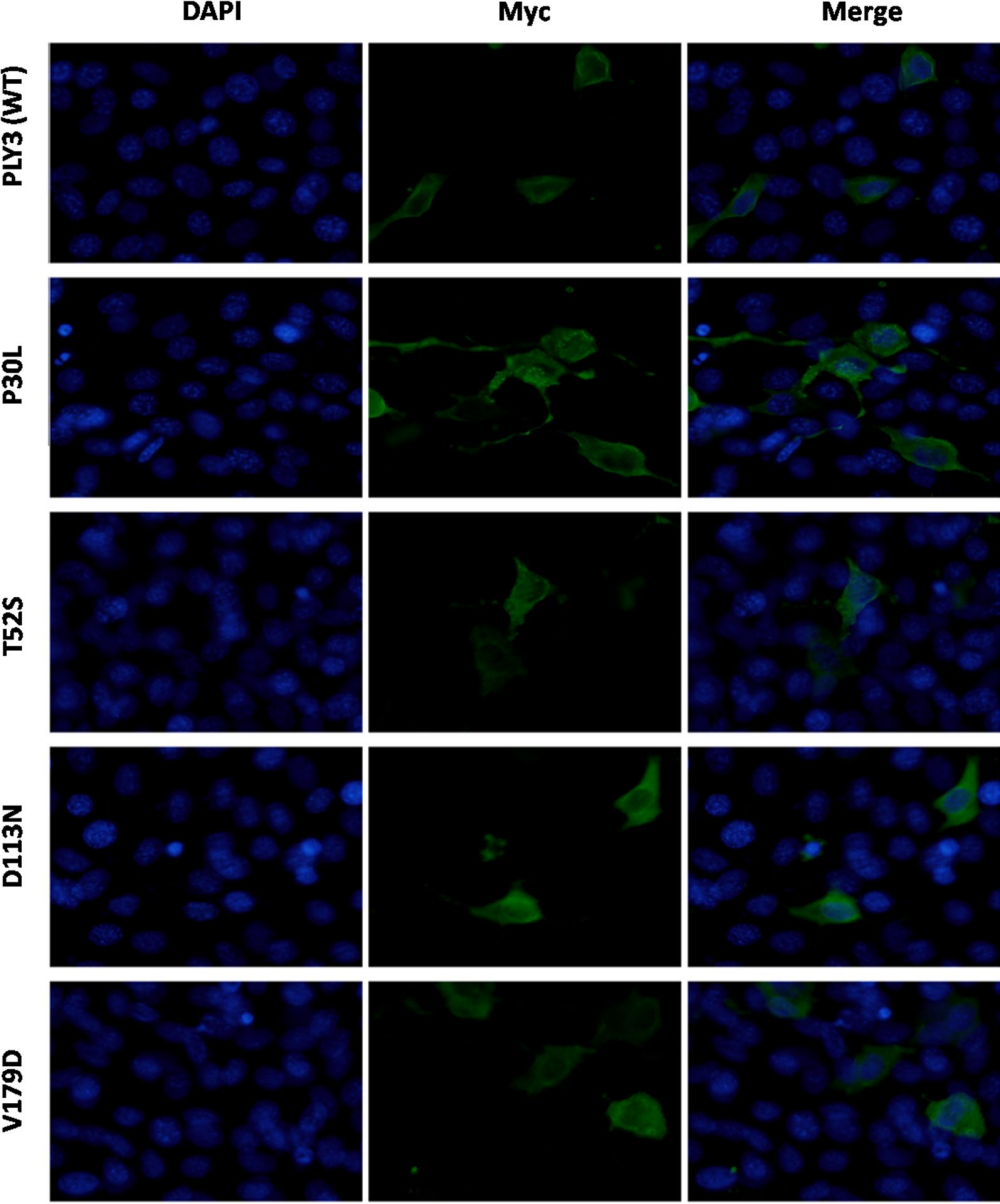
Subcellular localization of wild-type and variant RIPPLY3 proteins in the HEK293T cells. The wild-type and variant RIPPLY3 (P30L, T52S, D113N and V179D) were located in both cytoplasm and nucleus, and there seems no significant difference between them

The generated plasmids were transfected into HEK293T cells to investigate the effect of the variants on the trafficking of the RIPPLY3 protein. Immunofluorescence staining showed that both the wild-type RIPPLY3 and variant RIPPLY3 proteins (p.P30L, p.T52S, p.D113N and p.V179D) were located in both the cytoplasm and nucleus (Fig. 2). The TBX1 protein was located only in the nucleus (Additional file 1: Figure S1).

### The variants do not affect the expression of RIPPLY3

**Fig. 3:**
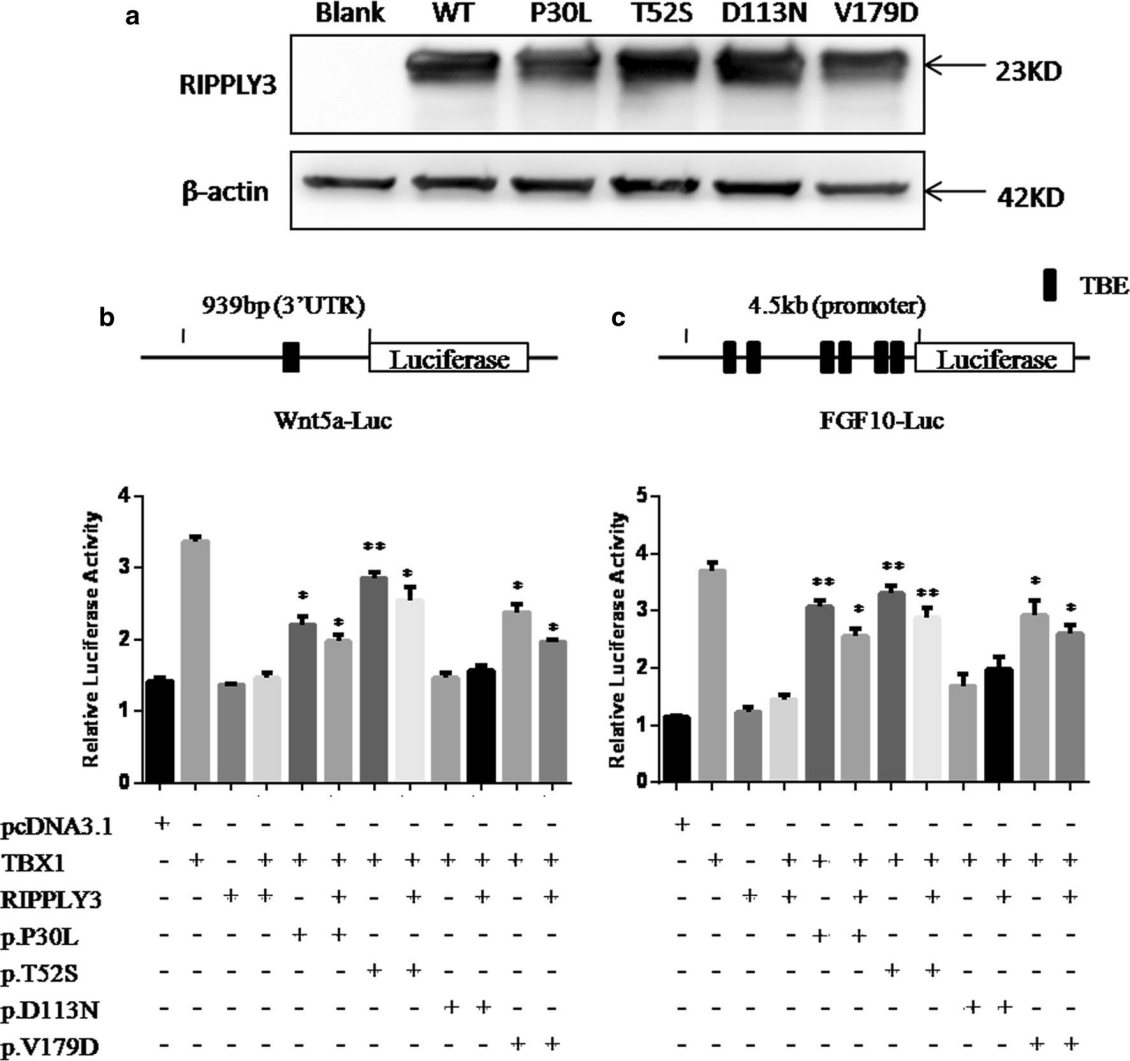
Western blot analysis of RIPPLY3 expression and functional analysis of the RIPPLY3 variants in the inhibition of TBX1 transcriptional activity in vitro. **a** Variant RIPPLY3 protein showed no significant change in dosage; **b**, **c** Wild-type TBX1 transactivated the wnt5a-Luc reporter and FGF10-Luc reporter compared with the empty expression vector, and wild-type RIPPLY3 showed inhibition of TBX1 transcriptional activity. Compared with wild-type RIPPLY3, three RIPPLY3 variants (p.P30L, p.T52S and p.V179D) showed impaired inhibition of TBX1 transcriptional activity in both homozygote and heterozygote. The results are expressed as relative luciferase activity and the presented values are the mean ± standard deviation of three independent experiments carried out in duplicates. *p < 0.05, **p < 0.01, when compared with wild type RIPPLY3

Wild-type and variant *RIPPLY3* expression plasmids were separately transfected into HEK293T cells. Western blot results revealed that there was no significant change in dosage between wild-type and variant RIPPLY3 proteins (Fig. 3a).

### The p.P30L, p.T52S and p.V179D variants show impaired inhibition of TBX1 transcriptional activity in vitro

RIPPLY3 is able to repress the transcriptional activity of TBX1 in vitro. To further validate the effects of these variants, we performed dual luciferase reporter assays using the *Wnt5a*-luc and *FGF10*-luc in HEK293T cells and C2C12 cells. All of the *RIPPLY3* variants are heterozygous in patients, so we also detected whether the variants would affect function of the wild-type RIPPLY3 protein by co-transfected the variant and wild-type RIPPLY3 protein together. Compared to the wild-type RIPPLY3 protein, the p.D113N variant showed no significant change in inhibition of TBX1 transcriptional activity in our assay. The others, namely, p.P30L, p.T52S and p.V179D, showed impaired inhibition of TBX1 transcriptional activity in both 293T cells for *Wnt5a*-luc (p.P30L: p = 0.0144 for homozygote and p = 0.0258 for heterozygote; p.T52S: p = 0.0031 for homozygote and p = 0.0167 for heterozygote; p.V179D: p = 0.0109 for homozygote and p = 0.0105 for heterozygote, respectively) and C2C12 cells for *FGF10*-luc. (p.P30L: p = 0.0032 for homozygote and p = 0.0102 for heterozygote; p.T52S: p = 0.0036 for homozygote and p = 0.0098 for heterozygote; p.V179D: p = 0.016 for homozygote and p = 0.0102 for heterozygote, respectively) (Fig. 3b, c). Therefore, the three variants (p.P30L, p.T52S and p.V179D) showed impaired inhibition of TBX1 transcriptional activity in both heterozygote and homozygote, although it seems that RIPPLY3 variants do not have significant dominant negative effect on the wild-type protein.

### The p.T52S variant affects the interaction of RIPPLY3 with TBX1

**Fig. 4:**
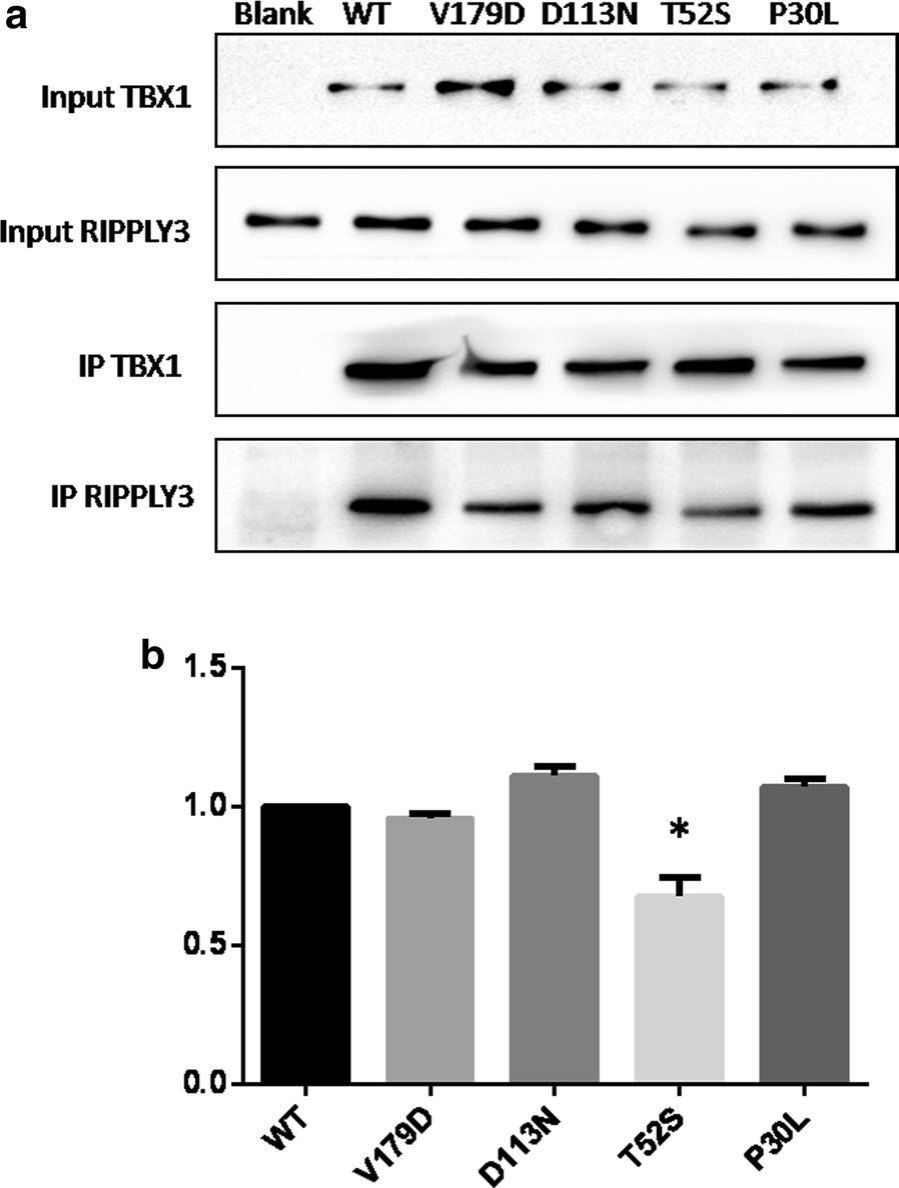
Co-immunoprecipitation assays to analysis the effect of RIPPLY3 variants on RIPPLY3-TBX1 interactions. **a** The whole cell extracts from the HEK293T cells overexpressing either wild-type or variant RIPPLY3 and TBX1 were immunoprecipitated using the anti-TBX1 antibody; **b** the grayscale value of the Co-IP protein bands was calculated using Image J software, the results are expressed as the ratio of the grayscale value of RIPPLY3 protein band divided by the TBX1, and the wild-type ratio is standardized to 1.0. Co-IP studies showed that the p.T52S significantly disrupted the physical interaction between RIPPLY3 and TBX1, whereas the p.P30L, p.D113N and p.V179D had no effect on the interaction between RIPPLY3 and TBX1 compared with the wild-type. *p < 0.05, when compared with wild type RIPPLY3

Evidence suggested that RIPPLY3 inhibited TBX1 transcriptional activity by physically interacting with TBX1 in a T-domain-dependent manner [15]. To characterize the effect of the variants on the protein–protein interaction, co-immunoprecipitation assays were performed using equal amounts of protein extracts from HEK293T cells overexpressing the wild-type and variant RIPPLY3 proteins. The results showed that the p.T52S variant significantly disrupted the physical interaction between RIPPLY3 and TBX1 (p = 0.0059). However, the p.P30L, p.D113N and p.V179D variants had no effect on the interaction between RIPPLY3 and TBX1 (Fig. 4).

## Discussion

Dysfunctional RIPPLY3 predisposing to congenital cardiovascular defects has been substantiated in animal models [15]. In mice, *Ripply3*^−/−^ embryos were born but died with cyanosis within 24 h after birth. In addition, an outstanding characteristic of *Ripply3*^−/−^ embryos is their almost complete lack of the third and fourth pharyngeal arches [15]. Therefore, *Ripply3*^−/−^ mouse embryos showed abnormal development of the vascular system, including misshapen major blood vessels and deletion of the aortic arch. In addition, an abnormality in cardiac outflow tract development was also seen in the *Ripply3*^−/−^ embryos, including hypotrophy of the aorta and incomplete formation of the ventricular septum [15]. The phenotypes partly resembled that of mouse embryos overexpressing *Tbx1* [21, 32]. Meanwhile, previous studies have shown that RIPPLY3 interacts with TBX1, and inhibits the transcriptional activity of TBX1 [14, 15]. Taken together, these experimental findings from animals have provided strong evidence that *RIPPLY3* plays a pivotal role in cardiovascular morphogenesis, especially in cardiac outflow tract development. Functionally compromised *RIPPLY3* may underlie a wide variety of congenital cardiac malformations, including CTD in humans.

In the present study, four rare, heterozygous and nonsynonymous variants of RIPPLY3—p.P30L, p.T52S, p.D113N and p.V179D—were identified in 577 unrelated CTD patients without the 22q11.2 deletion/duplication. The variant NM_018962.2:c.155C>G (p.T52S) is referred as rs745539198 with a 3.32e−05 allelic frequency, and the variant NM_018962.2:c.337G>A (p.D113N) is referred as rs747419773 with a 1.654e−05 allelic frequency. Interestingly, in spite of the very low allelic frequency, the p.T52S variant was only found in the Asian population, suggesting the presence of race specificity. However, variants p.P30L and p.V179D were not found in multiple online human gene variation databases. A cross-species alignment of multiple RIPPLY3 protein sequences showed that both the p.P30L and p.T52S occur in the highly evolutionarily conserved residues, while the p.D113N and p.V179D were not conserved. The p.T52S variant was predicted to be “possibly damaging” by the Polyphen2 software. Further functional analysis revealed that three of these variants, p.P30L, p.T52S, and p.V179D, showed impaired inhibition of TBX1 transcriptional activity in both heterozygote and homozygote, and the p.T52S variant affected the interaction of RIPPLY3 with TBX1 in vitro. Moreover, there were no deleterious variants identified in CHD pathogenic genes (*TBX1*, *GATA4*, *NKX2.5* and *TBX5*) in patients harboring RIPPLY3 variants.

The human *RIPPLY3* gene is composed of four exons that encode a protein of 190 amino acids and maps to chromosome 21q22.13.RIPPLY3 protein has two highly conserved regions found in all members of the Ripply family: a WRPW motif and a C-terminal Ripply homology domain (also called the bowline-DSCR-LedgerLine conserved ‘BDLC’ region) [33]. The WRPW motif (amino acids 39–42) facilitates interaction with GROUCHO [34, 35]. The ‘BDLC’ domain (amino acids 77–112) is predicted to contact with T-box proteins [36]. RIPPLY3 can recruit the Groucho/TLE co-repressor to TBX1 and control its intrinsic transcriptional activity. It has been established that TBX1 is an upstream regulator of select genes expressed during embryogenesis and cardiac morphogenesis, including the genes that encode Wnt family member 5A [27] and fibroblast growth factor 10 [28]. Therefore, the functional characteristics of the RIPPLY3 variants may be delineated by analyzing the inhibition of TBX1 transcriptional activity on the luciferase reporter Wnt5a-luc and FGF10-luc.

Dual luciferase reporter assays revealed that variants p.P30L, p.T52S and p.V179D, showed impaired inhibition of TBX1 transcriptional activity in both heterozygote and homozygote in vitro, and indicating that heterozygous of these variants could also be deleterious. It is intriguing that protein–protein interaction was hampered by the p.T52S variant, although this variant is not located in the ‘BDLC’ domain, which is predicted to contact with T-box proteins [36]. This may be ascribed to the distortion of the three-dimensional structure of the variant protein, hindering in such away its interaction with the TBX1 protein. The p.P30L and p.V179D variants also showed impaired inhibition of TBX1 transcriptional activity, however, these two variants seemed to have no effect on the physical interaction of TBX1 and RIPPLY3. This goes in parallel with previously reported variants in *GATA4 *and *MESP1* [37–39]. These variants reduced the transcriptional activity of GATA4 or MESP1 without affecting their affinity of binding to DNA or another protein partner, as is the case with the p.P30L and p.V179D variants. It is difficult to say whether the impaired inhibition of TBX1 transcriptional activity with these two variant RIPPLY3 proteins (p.P30L and p.V179D) is due to protein instability or the inability of recruiting other TBX1 partners, such as the Groucho/TLE, to modulate its activity, or a combination of both. Further studies are needed to clarify the specific mechanism.

Additionally, immunofluorescence staining revealed that the subcellular localization of these four variants was not affected. Although much is known about the interaction between RIPPLY3 and TBX1 in the pharyngeal apparatus and heart development [15], relatively less is known of RIPPLY3 playing a pivotal role in the development of other tissues, such as pre-placodal ectoderm and pancreas. Previously studies showed that RIPPLY3, acting downstream of retinoic acid receptor signaling, is a key player in establishing boundaries in the PPE [16]. RIPPLY3 negatively regulates the proliferation of early endocrine cells, acting as an Insm1-regulated gene enriched in the Pdx1-high cell population [40]. RIPPLY3 is also expressed in pancreatic β-cells, partially in the cytoplasm. Therefore, the subcellular localization of RIPPLY3 may not be limited to the nucleus.

This study has some limitations. Firstly, all functional assays in this study were performed in vitro, transgenic animal models would be helpful to confirm the potential function of these variants. Moreover, the specific mechanism of how the p.P30L and p.V179D variants impaired inhibition of TBX1 transcriptional activity need to be elucidated in the future.

## Conclusion

In conclusion, our study suggests that deleterious variants in *RIPPLY3* are potential molecular mechanisms involved in the pathogenesis of human CTD. This could have critical implications for clinical practice and genetic counseling. Furthermore, these findings reaffirm the importance of the cardiac transcription factor network and demonstrate that protein binding partners could be related to congenital heart disease. Additional studies that test a larger population and more extensive CHD phenotypes will help further our understanding of the role of *RIPPLY3* in CHD molecular pathogenesis.

## Additional file

**Additional file 1: Table S1.** Primer pairs used to amplify the coding regions contain candidate variants. Table S2. Primer pairs used to amplify the TBX1C variants identified in patients harboring RIPPLY3 variants. Figure S1. Subcellular localization of wild-type TBX1 protein in transiently transfected HEK293T cells. The wild-type TBX1 localized exclusively to the nuclei with normal nuclear distribution.

## Abbreviations

CTD: conotruncal heart defect
CHD: congenital heart disease
TOF: tetralogy of Fallot
TGA: transposition of the great arteries
DORV: double outlet of right ventricle
PTA: persistent truncus arteriosus
PA/VSD: pulmonary atresia with ventricular septal defect
IAA: interrupted aortic arch
MLPA: modified from the multiplex ligation-dependent probe amplification
CPELA: cycled primer extension and ligation-dependent amplification
TBEs: T-box binding sites
RT: room temperature
PDA: patent ductus arteriosus
PPT: pathogenicity prediction tool
CNV: copy number variant
SNP: single nucleotide polymorphism
