# Supplementary material for: A loss-of-function mutation p.T52S in RIPPLY3 is a potential predisposing genetic risk factor for Chinese Han conotruncal heart defect patients without the 22q11.2 deletion/duplication
Source: J Transl Med. 2018 Sep 21;16:260. doi: 10.1186/s12967-018-1633-1 (PMC6151064; doi:10.1186/s12967-018-1633-1)
Supplement: Supplementary file 1 — Additional file 1: Table S1. Primer pairs used to amplify the coding regions contain candidate variants. Table S2. Primer pairs used to amplify the TBX1C variants identified in patients harboring RIPPLY3 variants. Figure S1. Subcellular localization of wild-type TBX1 protein in transiently transfected HEK293T cells. The wild-type TBX1 localized exclusively to the nuclei with normal nuclear distribution. [file 12967_2018_1633_MOESM1_ESM.docx]

**Table S1. Primer pairs used to amplify the coding regions contain candidate variants**

| Candidate variants | Primer orientation | Primer sequences | Product size(bp) |
| --- | --- | --- | --- |
| c. 89C>T | F | 5’- TTTCTTTGTCCCGTCCCCACC-3’ | 613 |
|  | R | 5’- CGGCTCAGGAGCTTGCTTGG-3’ |  |
| c.155C>G | F | 5’-ATAGTTTGAAGGCAGCAGAAGT-3’ | 346 |
|  | R | 5’- TAGGAAGGAGAAGCCACCAG-3’ |  |
| c.337G>A | F | 5’- GCAAGCCACGATTGACTTCTA-3’ | 235 |
|  | R | 5’- ACCCCTTGAGGAGACACCTT-3’ |  |
| c.536T>A | F | 5’- AGACCAGGGCATCAACCAAG-3’ | 464 |
|  | R | 5’- GGTGGAGTGGGCGACTATCT-3’ |  |

**Table S2. Primer pairs used to amplify the *TBX1C* variants identified in patients harboring *RIPPLY3* variants.**

| *TBX1* variants  NM_080647.1 | Primer orientation | Primer sequences | Product size(bp) |
| --- | --- | --- | --- |
| c.1189A>C | F | 5’-CCAAGAGCCTTCTCTCCGC-3 | 775 |
|  | R | 5’-TGGGGAACCGGATACTTCGA-3’ |  |
| c.928G>A | F | 5’-GGTGCGCTTCTCCTAACACTC-3 | 722 |
|  | R | 5’-GGAGAGGGCCGAGGAGTG-3 |  |


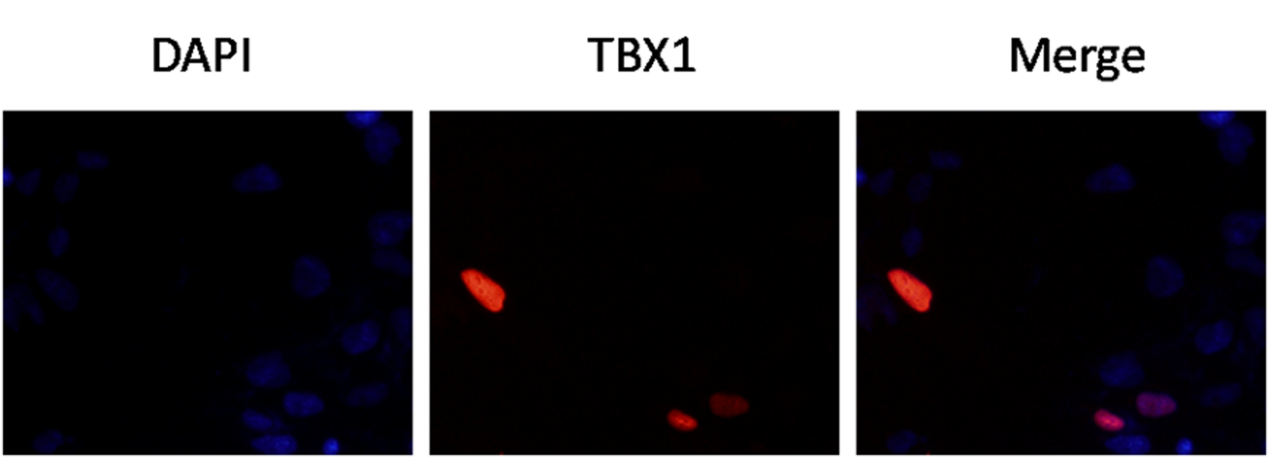


Figure S1. Subcellular localization of wild-type TBX1 protein in transiently transfected HEK293T cells. The wild-type TBX1 localized exclusively to the nuclei with normal nuclear distribution.
